# Elabela Attenuates the TGF-β1-Induced Epithelial-Mesenchymal Transition of Peritoneal Mesothelial Cells in Patients Receiving Peritoneal Dialysis

**DOI:** 10.3389/fphar.2022.890881

**Published:** 2022-06-21

**Authors:** Shunyun Xie, Feng Xu, Yue Lu, Yixian Zhang, Xinyang Li, Mengyuan Yu, Wenpeng Cui

**Affiliations:** Department of Nephrology, The Second Hospital of Jilin University, Changchun, China

**Keywords:** peritoneal fibrosis, apelin, HPMCs, EMT, chronic kidney disease, TGF-beta/SMAD/ERK/AKT pathway, elabela, peritoneal dialysis

## Abstract

Peritoneal fibrosis (PF), a common complication in patients receiving peritoneal dialysis (PD), is primarily caused by the epithelial-mesenchymal transition (EMT) of human peritoneal mesothelial cells (HPMCs). PF is the main reason for patients on PD to withdraw from PD. Effective treatment is unavailable for this complication at present. Elabela (ELA) is a polypeptide hormone secreted by the vascular endothelium and kidney. Peptide hormones ELA and apelin (APLN) have various protective effects on the cardiovascular and urinary systems and have potential therapeutic effects on organ fibrosis. ELA and APLN are less studied in PD population. Here, we aimed to investigate the clinical significance of ELA in patients on PD and to evaluate the therapeutic effect of ELA on EMT of HPMCs. Compared with those in patients with stage 5 chronic kidney disease who are not on dialysis, serum ELA levels in patients on PD increased with the improvement of residual renal function at PD duration <36 months and decreased to pre-dialysis levels at PD duration ≥36 months, suggesting that dialysis duration is the main risk factor affecting serum ELA levels in patients on PD. In addition, serum APLN levels decreased in the early stage of PD and recovered to the pre-dialysis level with the prolongation of dialysis time. Notably, serum APLN levels were positively correlated with dialysis duration in patients undergoing PD. To establish the EMT model, we stimulated HPMCs using transforming growth factor-beta 1 (TGF-β1) in cell experiments performed *in vitro*. ELA-32 treatment reversed the TGF-β1-induced reduction in the expression of the epithelial cell marker and suppressed the expression of mesenchymal cell markers by inhibiting the phosphorylation of SMAD2/3, ERK1/2, and AKT. Therefore, our findings imply that ELA-32 can interfere with the EMT of HPMCs by inhibiting the activation of the TGF-β/SMAD2/3, ERK1/2, and AKT pathways, providing novel insights on the potential therapeutic use of ELA for treating PD-related PF.

## Introduction

As one of the most common renal replacement therapies for patients with stage 5 chronic kidney disease (CKD5), peritoneal dialysis (PD) is more effective than hemodialysis in maintaining residual renal function, hemodynamic stability, and improving quality of life (Mehrotra et al., 2016). PD uses the peritoneal tissue to remove metabolic waste and excess fluid from the body. Peritoneal function often affects the duration of treatment in patients receiving PD treatment. The main cause of peritoneal dysfunction is peritoneal fibrosis (PF), which is caused by various factors including the stimulation of non-physiological peritoneal dialysate and increased glycosylation product formation and TGF-β1 secretion (Balzer, 2020). The clinicopathological manifestations of PF include the epithelial-mesenchymal transition (EMT) of human peritoneal mesothelial cells (HPMCs), submesothelial thickening, increased blood vessel formation, and elevated autocrine activity. EMT plays a central role in initiating and accelerating PF (Selgas et al., 2006). The above changes eventually lead to ultrafiltration failure and decrease in dialysis adequacy when patients receive PD treatment, which, in severe cases, cause patients to withdraw from PD (Aroeira et al., 2007). Currently, effective treatment for PF is unavailable in clinical settings.

For treatment of PF, the addition of peptides showed a significant therapeutic effect on the treatment of PD-related PF. For example, Ferrantelli et al. (2016) reported that the intraperitoneal administration of alanyl-glutamine can reduce peritoneal thickness, alpha-smooth muscle actin (α-SMA) expression, and local angiogenesis by regulating the expression of the inflammatory cytokine interleukin (IL)-17. In addition, the use of peptides can attenuate the production of protein glycosylation products (Alhamdani et al., 2007), which contributes to protection of the peritoneal structure (Honda et al., 1999). However, no peptides have yet been successfully applied in clinical treatment. Therefore, we sought to identify a similar polypeptide that could play a role in inhibiting PF.

APJ is a G protein-coupled receptor with 30% homology to angiotensin receptor 1 (O’Dowd et al., 1993). Apelin (APLN) is the first discovered ligand of the APJ receptor (Tatemoto et al., 1998). Based on its distribution level in the body, APLN is commonly regarded as a fat factor because its content in the surrounding tissue is significantly higher than that in the serum (Boucher et al., 2005). Elabela (ELA) was discovered as the second endogenous ligand of APJ in 2013 (Chng et al., 2013). The ELA gene, located in the autosomal chromosome, transcribes and translates into a 54-amino acid precursor polypeptide with a signal peptide fragment, and its active end product is ELA-32 peptide. The ELA-32 peptide chain has two potential protease cleavage sites, which can be further metabolized to ELA-21 and ELA-11 (Yang et al., 2017). The three peptides have similar biological effects, but mainly, ELA-32 exhibits biological activity and has a longer half-life in terms of metabolism (Nyimanu et al., 2021). In adulthood, ELA is mainly expressed in the human heart, vascular endothelial cells, and kidneys (Wang et al., 2015). The axis composed of APLN, ELA, and APJ receptors has shown beneficial effects in the pathophysiological process of cardiovascular diseases and renal diseases (Chapman et al., 2021). The ELA/APLN-APJ axis is involved in regulating the metabolism of vascular endothelial cells and renal tubular epithelial cells under various pathological conditions, such as ischemia and hypoxia, and has shown good anti-EMT performance. However, the regulatory mechanisms by which it exerts protective effects are complex and vary in different pathological conditions.

The serum levels of APLN and ELA in the human body are affected by renal function (Dogan et al., 2018; Lu et al., 2020). Moreover, in patients undergoing PD, the changes in serum levels of ELA and APLN and factors influencing these changes remain unclear. Therefore, we detected the serum levels of ELA and APLN in patients on PD to explore the connection between the ligands and PD. We further studied whether ELA has an anti-EMT effect on HPMCs stimulated *in vitro* and its therapeutic potential when added to the peritoneal dialysate for PF treatment.

## Materials and Methods

### Study Subjects

From September 2020 to September 2021, patients with CKD5 who were on PD and patients with CKD5 who were not on dialysis admitted to the Department of Nephrology, the Second Hospital of Jilin University (Changchun, China) were screened. The inclusion criteria were as follows: 1) patients with PD as the only renal replacement therapy; and 2) patients with CKD5 who had not received renal replacement therapy. The exclusion criteria were as follows: 1) patients aged ≤18 years; 2) patients undergoing regular hemodialysis combined with PD; 3) patients with history of abdominal surgery in the past 2 weeks; 4) patients with severe heart failure and/or brain natriuretic peptide (BNP) levels >500 pg/ml; 5) patients with pulmonary infection, peritonitis, and other infectious diseases; 6) patients with severe liver diseases, hematological diseases, malignant tumors, and other consumptive diseases affecting the whole body; and 7) patients with incomplete or no clinical data. In total, we collected fasting serum samples from 20 patients with CKD5 and 60 patients on PD. Among them, the patients on PD were divided into long-term and short-term dialysis groups based on whether the treatment time exceeded 36 months. Furthermore, patients were divided into three groups: CKD5 group, eGFR < 15 (ml/min/1.73 m^2^) without renal replacement therapy; PD < 36 group, patients who received PD with PD treatment time <36 months; and PD ≥ 36 group, patients who received PD with PD treatment time ≥36 months. Informed consent was obtained from the subjects before the collection of all serum samples; the study was approved by the Ethics Committee of the Second Hospital of Jilin University (approval number: 2020010).

### Clinical Data Collection

The age, sex, body mass index (BMI), dialysis duration, primary disease, history of hypertension and diabetes, systolic and diastolic blood pressure, residual urine volume, 24 h ultrafiltration volume of peritoneal dialysate, and routine laboratory indicators of the patients on PD were collected. The residual glomerular filtration rate (rGFR) in the patients on PD was calculated using the following formula: (Qin et al., 2020).

rGFR=(residual creatine clearance+residual urea clearance)/2

### Enzyme-Linked Immunosorbent Assay (ELISA)

Fasting venous blood samples were collected from the patients on PD and patients with CKD5 in the morning and placed in a vacuum tube filled with separation gel-coagulant. The upper serum was extracted *via* centrifugation at 3,000 rpm for 10 min at 4°C and frozen in the refrigerator at −80°C. Human ELA Elisa Kit (S-1508; Peninsula Laboratories International, BMA Biomedicals, Basel, Switzerland) and human APLN Elisa Kit (E-EL-H0456C; Elabscience, Wuhan, China) were used to determine the serum ELA and APLN levels, respectively.

### Chemicals

ELA-32 (amino acid sequence: QRPVNLTMRRKLRKHNCLQRRCMPLHSRVPFP) was synthesized by GenStar (Beijing, China) and stored at −20°C. The ELA-32 powder was dissolved in aseptic deionized water to the cryopreservation concentration, sub-packed, and stored at −80°C.

### Cell Culture and Treatment

The immortalized HPMC line HMrSv5 (Jennio Biotech Co., Ltd., Guangzhou, China) was cultured in high-glucose (4.5 g/L) Dulbecco’s Modified Eagle Medium (DMEM; Gibco, Thermo Fisher Scientific, Waltham, MA, United States) supplemented with 10% fetal bovine serum (FBS; Gibco) and incubated at 37°C and 5% CO_2_. All experiments were performed after 24 h of treatment using serum-free media. To induce EMT, cells were treated with 10 ng/ml TGF-β1 (cat:100-21, Peprotech, Rocky Hill, CT, United States) for 24 h. HMrSv5 was divided into four groups according to different treatments: 1) Control group where cells were cultured with DMEM medium containing 10% FBS after 24 h of synchronization; 2) ELA group where cells were treated with ELA-32 (10 μM) for 24 h; 3) TGF-β1 group where cells were treated with TGF-β1 (10 ng/ml) for 24 h; and TGF-β1 + ELA group where cells were co-treated with TGF-β1 (10 ng/ml) and ELA-32 (10 μM) for 24 h.

### Transwell Assay

The HPMCs in logarithmic growth phase were subjected to TGF-β1 (10 ng/ml) treatment with or without ELA-32 for 24 h. After stimulation, the cells were digested with trypsin, and the sample was centrifuged (1,000 rpm, 5 min). The supernatant was discarded, and the cells were resuscitated with serum-free DMEM. The number of terminal cells was adjusted to 5 × 10^5^/ml. DMEM (800 μl) containing 10% FBS was added to the lower chamber of the 24-well plate; a 200-μl cell suspension was added to the upper chamber, and the culture was maintained in the aseptic cell incubator for 24 h. The chamber was taken out, and the cells were washed with 1 × phosphate buffered saline (PBS, 548117; Sangon Biotech, Shanghai, China), fixed with 4% paraformaldehyde, and stained with 0.1% crystal violet. The chambers were dried and analyzed using an inverted microscope. The average count from the fields was calculated, and the experiments were independently repeated four times.

### Wounding Healing Assay

The HPMCs in the logarithmic growth phase were subcultured in a 6-well plate and cultured in DMEM containing 10% FBS when the cell fusion rate reached 100%. A 200-μl sterile liquid transfer gun head was used to scratch the cells perpendicular to the cell plane and to the line marked in advance at the back of the plate. The cells were washed thrice with aseptic 1 × PBS (B548117; Sangon Biotech), and the remaining non-adherent cells were washed out. The medium was replaced with serum-free DMEM and treated with or without TGF-β1 and ELA-32 for 24 h. Before and after the experiment, the scratch width was measured, and the cells were observed under a light microscope. The experiments were independently repeated four times.

### Immunofluorescence Assay

The HPMCs (1 × 10^5^/ml) were inoculated in 25-mm aseptic cell climbing tablets, cultured for 24 h, and synchronized with serum-free DMEM for 24 h. The cells were treated TGF-β1 (10 ng/ml) with or without ELA-32 for 24 h. After stimulation,the cells were washed with 1 × PBS (B548117; Sangon Biotech). Then, the cells were fixed with 4% paraformaldehyde, incubated with 0.1% Triton X-100 for 15 min at 27°C, and blocked with goat serum for 30 min. The primary antibodies of α-SMA (ab7817; Abcam), E-cadherin [14472; Cell Signaling Technology (CST), Danvers, MA, United States], and fibronectin (ab45688; Abcam) diluted to 1:200 in PBS were added and covered on the cell slides, and incubated overnight at 4°C. After washing with 1 × PBS (5 min, 3 times), the cells were incubated with Anti-Rabbit IgG (P0176, Beyotime Biotechnology, Jiangsu, China) for 1 h at 27°C in the dark. Finally, the cells were washed with 1 × PBS in the dark (5 min, 3 times), mounted on a sealing tablet containing 4′,6-diamidino-2-phenylindole (DAPI; ab104139; Abcam, Cambridge, UK), and observed under a laser scanning confocal microscope (Olympus, Japan). For the fluorescence results, mean immunofluorescence intensity measurements on cell climbing tablets were performed using ImageJ (National Institutes of Health, Bethesda, MD, United States).

### Western Blot Analysis

The cells were incubated in radioimmunoprecipitation assay cleavage buffer (P0013B; Beyotime) containing 0.1 mM phenylmethylsulfonyl fluoride. The lysate was centrifuged, and the supernatant was collected. The protein concentration was determined using BCA Protein Detection Kit (P0010; Beyotime). The protein was resolved and separated on a gel, with a mass of 20 μg per lane. The extracted cleavage products were transferred to a PVDF membrane *via* sodium dodecyl sulfate–polyacrylamide gel electrophoresis and electroporation. After blocking with 5% milk in tris buffered saline with Tween (A100777; Sangon Biotech), the sample was incubated with the first antibody overnight at 4°C and then with the HRP-coupled anti-mouse/rabbit IgG secondary antibody for 1 h. After soaking in HRP Substrate (Millipore, Burlington, MA, United States) hypersensitive to electro-chemi-luminescence, the protein bands were observed using ChemiDoc™ MP Imaging System (Bio-Rad Laboratories, Hercules, CA, United States). The following antibodies were used for immunoblotting: α-SMA (ab7817; Abcam), fibronectin (ab45688; Abcam), E-cadherin (14472; CST), vimentin (5741; CST), p-SMAD2/3 (8828; CST), SMAD2/3 (8685; CST), AKT (AF6261; Affinity Biosciences, Cincinnati, OH, United States), p-AKT (AF0016; Affinity), ERK1/2 (4695; CST), p-ERK1/2 (9101S; CST), GAPDH (D190090; Sangon Biotech, Shanghai, China), HRP-coupled rabbit secondary antibody (ZB-2301; ZSGB-Bio, Beijing, China), and HRP-coupled anti-mouse secondary antibody (ZB-2305; ZSGB-Bio).

### Statistical Analysis

The data were analyzed using SPSS 25.0 statistical software (SPSS Inc., Chicago, IL, United States). For data with normal or approximately normal distribution (presented as mean ± SD), Student’s *t*-test was used to compare two groups, whereas single-factor analysis of variance (ANOVA) was used to compare among groups. For comparison among more than two groups and between any two groups, Mann–Whitney and Kruskal–Wallis tests, respectively, were used. The adoption rates or constituent ratio of the count data was also expressed, and chi-square test or Fisher exact probability test was performed to compare among groups. Finally, Pearson correlation, Spearman correlation, and multiple linear regression analyses were conducted to determine the correlation between variables. Differences with *p* < 0.05 were statistically significant.

## Results

### Serum Elabela and Apelin Levels Significantly Correlate With Dialysis Duration

In total, 20, 40, and 20 patients were included in the CKD5 group, the PD < 36 group, and the PD ≥ 36 group, respectively. In this study, no significant differences in age, history of hypertension and diabetes, primary glomerular diseases, and blood pressure were found in the CKD5 group, the PD < 36 group, or the PD ≥ 36 group. The dialysis duration of the PD < 36 group and PD ≥ 36 group was 9.00 (3.25, 18.75) and 49.50 (37.25, 70.50) months, respectively (Table 1). The three groups were analyzed based on the clinical laboratory tests of the serum. Compared with CKD5 group, the patients in PD < 36 group exhibited significantly higher levels of hemoglobin (Hb) and C-reactive protein (CRP), glomerular filtration rate (GFR), and carbon dioxide binding capacity (CO_2_P) (*p* < 0.05) (Table 1); levels of CRP, serum creatinine (Cre), and β2 microglobulin were further increased in PD ≥ 36 group (*p* < 0.05). However, no differences in the levels of Hb, blood urea nitrogen (BUN), and GFR were found between the CKD5 group and PD ≥ 36 group. In addition, no differences in the levels of serum albumin, parathyroid hormone, fasting venous blood glucose, and BNP among the three groups were observed (Table 1).

**TABLE 1:** Individual baseline characteristics in different groups.

	CKD5 group (*n* = 20)	PD < 36 group (*n* = 40)	PD ≥ 36 group (*n* = 20)	*p*-value
Age (year)	47.95 ± 15.54	44.98 ± 12.59	45.80 ± 9.11	0.763
Sex (male/female)	9/11	28/12	8/12	0.044
BMI (kg/m^2^)	24.06 ± 2.92	24.04 ± 3.74	23.98 ± 2.65	0.996
Hypertension, n (%)	17 (85)	38 (95)	20 (100)	0.132
Diabetes, n (%)	5 (25)	8 (20)	3 (15)	0.732
SBP (mmHg)	147.75 ± 19.79	148.99 ± 12.95	146.53 ± 24.18	0.899
DBP (mmHg)	88.85 ± 11.93	94.70 ± 11.26	91.89 ± 13.92	0.211
PD duration(month)	—	9.00 (3.25, 18.75)	49.50 (37.25, 70.50)	<0.001
Primary renal disease, n (%)				0.611
Glomerulonephritis	11 (55)	23 (57.5)	12 (60)	
Diabetic nephropathy	5 (25)	7 (17.5)	2 (10)	
Interstitial nephropathy	4 (20)	10 (25)	5 (25)	
Others	0 (0)	0 (0)	1 (5)	
Laboratory data				
Hb (g/L)	94.60 ± 19.30	111.03 ± 17.71^a^	95.55 ± 15.94^b^	0.001
Albumin (g/L)	37.74 ± 5.72	38.59 ± 3.81	35.73 ± 4.94	0.148
CO_2_P (mM)	19.31 ± 2.86	24.01 ± 4.11^a^	26.31 ± 4.45^a^	<0.001
BUN (mM)	27.96 ± 13.06	19.40 ± 5.73^a^	21.13 ± 4.47	0.024
Cre (μM)	788.65 ± 353.33	920.46 ± 360.13	1,134.70 ± 260.94^a^ ^,^ ^b^	0.003
β2-MG (mg/L)	16.59 ± 4.99	25.75 ± 8.48^a^	33.45 ± 6.55^a^ ^,^ ^b^	<0.001
eGFR(ml/min/1.73m^2^)/rGFR(ml/min)	6.56 ± 3.13	14.44 (5.13, 36.13)^a^	2.37 (0.10, 5.25)^a^ ^,^ ^b^	<0.001
PTH (pg/ml)	390.35 (256.78, 452.13)	392.00 (180.80, 533.13)	320.90 (168.20, 652.75)	0.985
CRP (mg/L)	1.37 (0.34, 2.39)	4.32 (2.22, 7.58)^a^	3.84 (1.15, 11.48)^a^	0.001
FBG (mM)	5.44 ± 1.29	6.35 ± 1.53	6.29 ± 3.06	0.222
BNP (ng/ml)	104.50 (20.50, 313.75)	112.50 (67.00,246.25)	191.00 (56.25, 464.00)	0.314
Dialysis parameters				
Peritoneal Kt/V	—	1.42 ± 0.49	1.54 ± 0.35	0.350
Renal Kt/V	—	0.57 (0.22, 1.00)	0.09 (0.01, 0.17)	<0.001
Total Kt/V	—	2.10 ± 0.79	1.79 ± 0.22	0.097
4h D/P	—	0.61 ± 0.16	0.64 ± 0.09	0.456
4h D/Do	—	0.45 ± 0.28	0.41 ± 0.08	0.577

ELA, elabela; CKD5, stage 5 chronic kidney disease; PD, peritoneal dialysis; BMI, body mass index; SBP, systolic blood pressure; DBP, diastolic blood pressure; Hb, hemoglobin; CO_2_P, carbon dioxide binding capacity; BUN, blood urine nitrogen; β2-MG, β2-microglobulin; Cre, serum creatinine; eGFR, estimated glomerular filtration rate; PTH, parathyroid hormone; rGFR, residual glomerular filtration rate; CRP, c-reaction protein; FBG, fasting blood glucose; BNP, brain natriuretic peptide; Kt/V, urea removal index; D/P, dialysate-to-plasma creatinine; D/Do, glucose uptake ratio. Data are expressed as mean ± SD.

a *p* < 0.05, vs. the CKD5 group.

b *p* <0.05, vs. the PD < 36 group.

**FIGURE 1:**
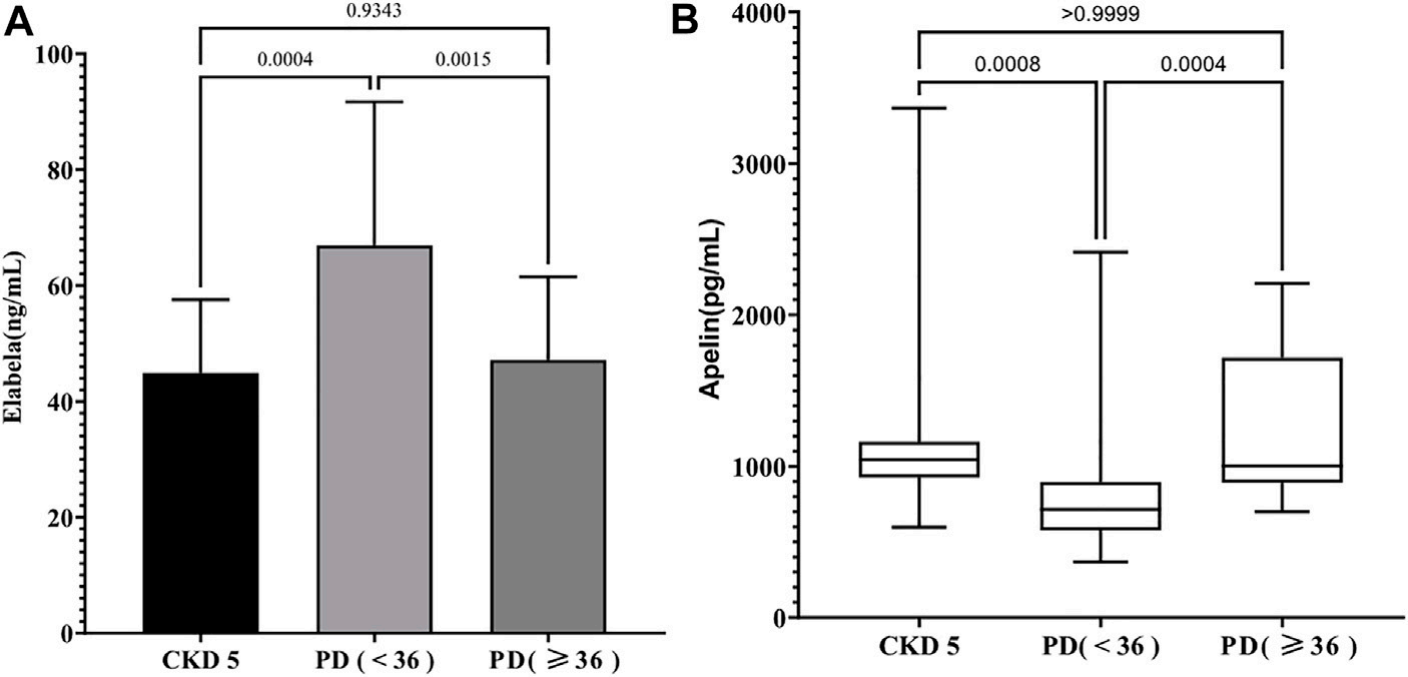
**(A)** The level of serum ELA in different groups; **(B)** The level of serum Apelin in different groups.

Compared with serum ELA levels of patients in the CKD5 group (44.92 ± 12.61 ng/ml), that of patients in the PD < 36 group was noted to be significantly higher (66.95 ± 24.73 ng/ml) (*p* < 0.05), but no significant difference (47.14 ± 14.34 ng/ml) (*p* > 0.05) was observed in that of patients in the PD ≥ 36 group (Figure 1A). In contrast, serum APLN levels of the patients in PD < 36 group [717.10 (575.95, 896.38) pg/ml] were decreased compared with that in the CKD5 group [1,042.84 (927.02, 1,164.62) pg/ml], but there were no similar differences between the CKD5 group and PD ≥ 36 group [1,003.09 (890.24, 1,718.88) pg/ml] (*p* > 0.05) (Figure 1B). In summary, serum ELA and APLN levels were different in PD patients at different periods.

### Dialysis Duration is the Risk Factor Influencing Serum Elabela Levels in Patients on Peritoneal Dialysis

To further analyze the effects of serum ELA and APLN in patients on PD, Spearman correlation analysis was performed using the clinical data obtained. We included the PD duration, age, and levels of Hb, CO_2_P, BUN, Cre, rGFR, BNP, CRP, and serum ELA and APLN levels for the correlation analysis. The results showed a negative correlation of serum ELA level with dialysis duration (*r* = −0.525, *p* < 0.05) and a positive correlation between serum ELA level and rGFR (*r* = 0.322, *p* < 0.05). At the same time, serum APLN level positively correlated with dialysis duration (*r* = 0.355, *p* < 0.05) (Table 2). Multiple linear regression was not possible because APLN positively correlated only with dialysis duration. After PD, an irreversible loss of renal function is commonly observed in patients. To determine the specific factors affecting the serum ELA level in patients on PD, stepwise multiple linear regression analysis was conducted using serum ELA concentration as the dependent variable. Notably, the results revealed that dialysis duration was the major risk factor affecting serum ELA levels in patients on PD (*r* = −0.412, *p* < 0.05) (Table 3).

**TABLE 2:** *R*-values and *p*-values of correlations among the study variables.

	ELA	Apelin	PD duration	Age	Hb	CO_2_P	BUN	Cre	rGFR	BNP
Apelin	−0.193									
	0.139									
PD	−0.525	0.355								
Duration	<0.001	0.005								
Age	−0.001	0.069	0.087							
	0.996	0.602	0.509							
Hb	0.148	−0.019	−0.249	−0.059						
	0.261	0.886	0.055	0.655						
CO_2_P	−0.010	0.198	0.032	−0.263	0.116					
	0.941	0.129	0.808	0.042	0.379					
BUN	0.013	−0.078	0.120	0.157	−0.183	−0.028				
	0.920	0.552	0.361	0.232	0.162	0.830				
Cre	−0.286	0.003	0.407	0.004	−0.345	0.041	0.500			
	0.027	0.984	0.001	0.977	0.007	0.754	<0.001			
rGFR	0.322	−0.173	−0.622	−0.048	0.352	−0.036	−0.223	−0.755		
	0.012	0.187	<0.001	0.715	0.006	0.786	0.086	<0.001		
BNP	−0.129	−0.006	0.033	0.187	−0.327	0.004	0.128	−0.022	−0.061	
	0.327	0.967	0.802	0.153	0.011	0.978	0.330	0.867	0.643	
CRP	0.070	0.037	−0.051	−0.050	−0.161	−0.152	−0.042	0.126	−0.258	0.240
	0.593	0.781	0.699	0.706	0.219	0.248	0.750	0.336	0.047	0.062

ELA, elabela; Hb, hemoglobin; PD, peritoneal dialysis; CO_2_P, carbon dioxide combining power; BUN, blood urine nitrogen; Cre, serum creatinine; rGFR, residual glomerular filtration rate; BNP, brain natriuretic peptide; CRP, c-reaction protein. The first row for each variable represents the *r* value, and the second row represents the *p* value.

**TABLE 3:** Stepwise multiple linear regression analysis of ELA.

Variables	Partial regression coefficient	SE	Standard partial regression coefficient	*t*	*p*	95% CI of partial regression coefficient
Constant	70.985	4.000		17.744	0.001	(62.977, 78.993)
PD duration	−0.412	0.112	−0.436	−3.687	0.001	(−0.636, −0.188)

ELA, elabela; SE, standard error; CI, confidence interval. Multiple R-squared: 0.190; adjusted R-squared: 0.176.

### ELA-32 Reduces TGF-β1-Induced Epithelial-Mesenchymal Transition of Human Peritoneal Mesothelial Cells

**FIGURE 2:**
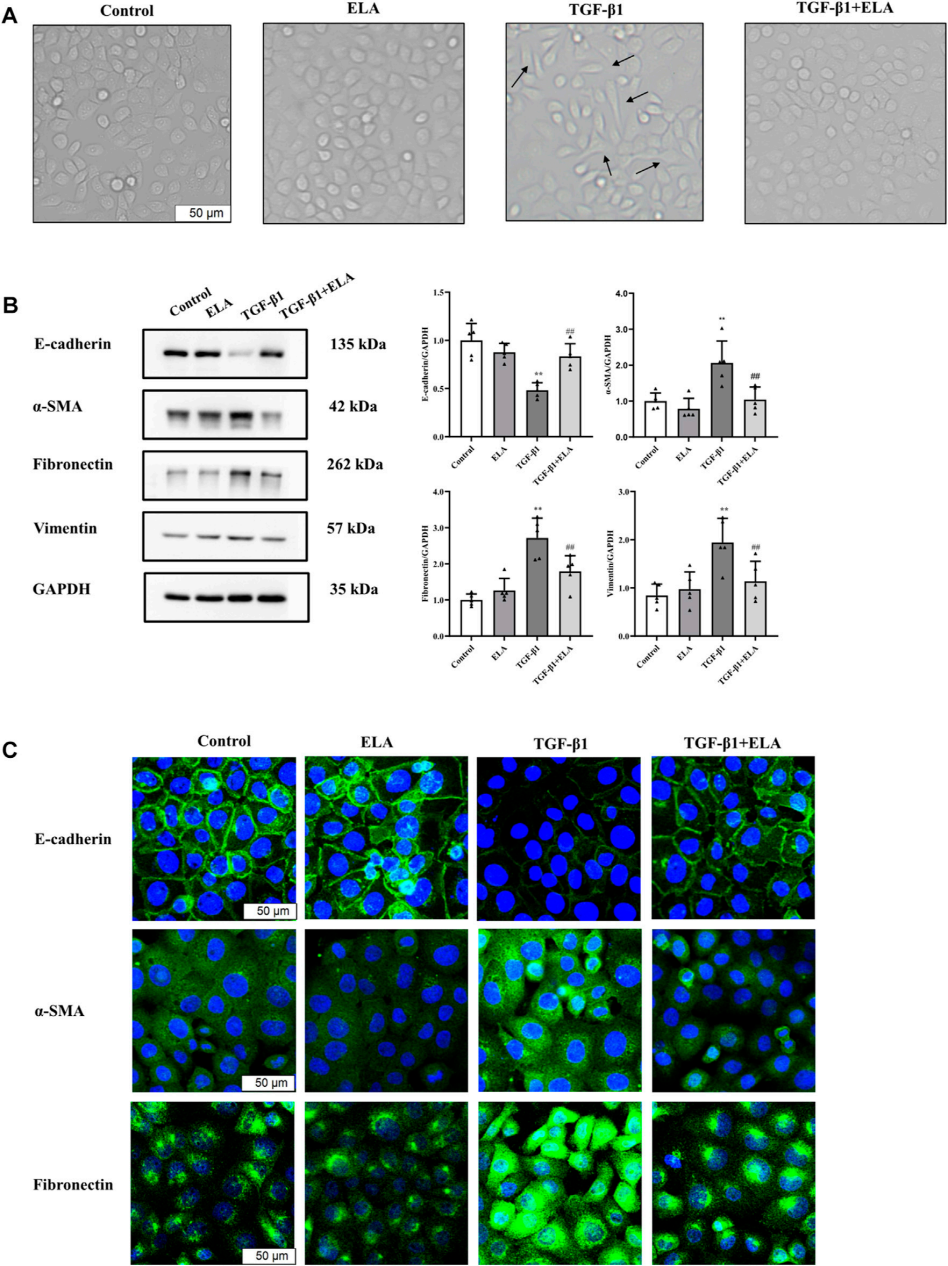
The role of ELA in TGF-β1-induced EMT in HMrSv5. HMrSv5 was divided into four groups according to different treatments: 1) Control group where cells were cultured with DMEM medium containing 10% FBS after 24 h of synchronization; 2) ELA group where cells were treated with ELA-32 (10 μM) for 24 h; 3) TGF-β1 group where cells were treated with TGF-β1 (10 ng/ml) for 24 h; and TGF-β1+ELA group where cells were co-treated with TGF-β1 (10 ng/ml) and ELA-32 (10 μM) for 24 h. Notes **(A)** Light microscope was used to observe the morphological changes of human peritoneal mesenchymal cells. **(B)** The expression of E-cadherin, α-SMA, fibronectin, and vimentin proteins were detected by Western blotting. The experiments were repeated five times. **(C)** The E-cadherin, α-SMA, Fibronectin were observed by immunofluorescence; cell nuclei were stained with DAPI (blue fluorescence) (scale bar = 50 μm). In HMrSv5, E-cadherin is a green fluorescence located in the cell membrane, α-SMA, fibronectin is a green fluorescence distributed in the cytoplasm. Data are expressed as mean ± SD, **p < 0.05 vs. control group; ^##^ *p* < 0.05 vs. TGF-β1 group. Abbreviations: ELA, elabela; TGF-β1, transforming growth factor-β1.

PMCs are one of the main components of the peritoneal tissue (Selgas et al., 2006). To determine whether ELA-32 holds significance in PD when added to the peritoneal dialysate, we established the EMT model using TGF-β1 (10 ng/ml) and treated the HPMCs with ELA-32 (10 μM). After 24 h, we discovered that compared with untreated cells (control group), the TGF-β1-group HPMCs lost their pebble-like appearance and became elongated spindles. Notably, ELA-32 treatment reduced the proportion of spindle cells, suggesting that the combined treatment with ELA-32 and TGF-β1 can inhibit the morphological changes induced by the previous TGF-β1 treatment (Figure 2A). Based on the Western blot assay, the TGF-β1 treatment significantly upregulated the expression of mesenchymal cell markers α-SMA, fibronectin, and vimentin and downregulated the expression of the epithelial cell marker E-cadherin. However, the combined treatment with ELA-32 and TGF-β1 significantly inhibited the expression of mesenchymal protein markers and restored the expression of E-cadherin (Figure 2B). To further confirm the anti-EMT effect of ELA-32, immunofluorescence staining was performed using E-cadherin, α-SMA, and fibronectin. Compared with the control group, the intracellular distribution of α-SMA and fibronectin in the TGF-β1 group increased, when observed under confocal fluorescence microscope, while the content of the protein E-cadherin decreased in the cell membrane; however, after ELA-32 treatment, the expression of the three markers were restored (Figure 2C). Taken together, these results suggest that ELA-32 inhibited HMrSv5 EMT induced by TGF-β1.

### ELA-32 Attenuates the TGF-β1-Induced Migration of Human Peritoneal Mesothelial Cells

**FIGURE 3:**
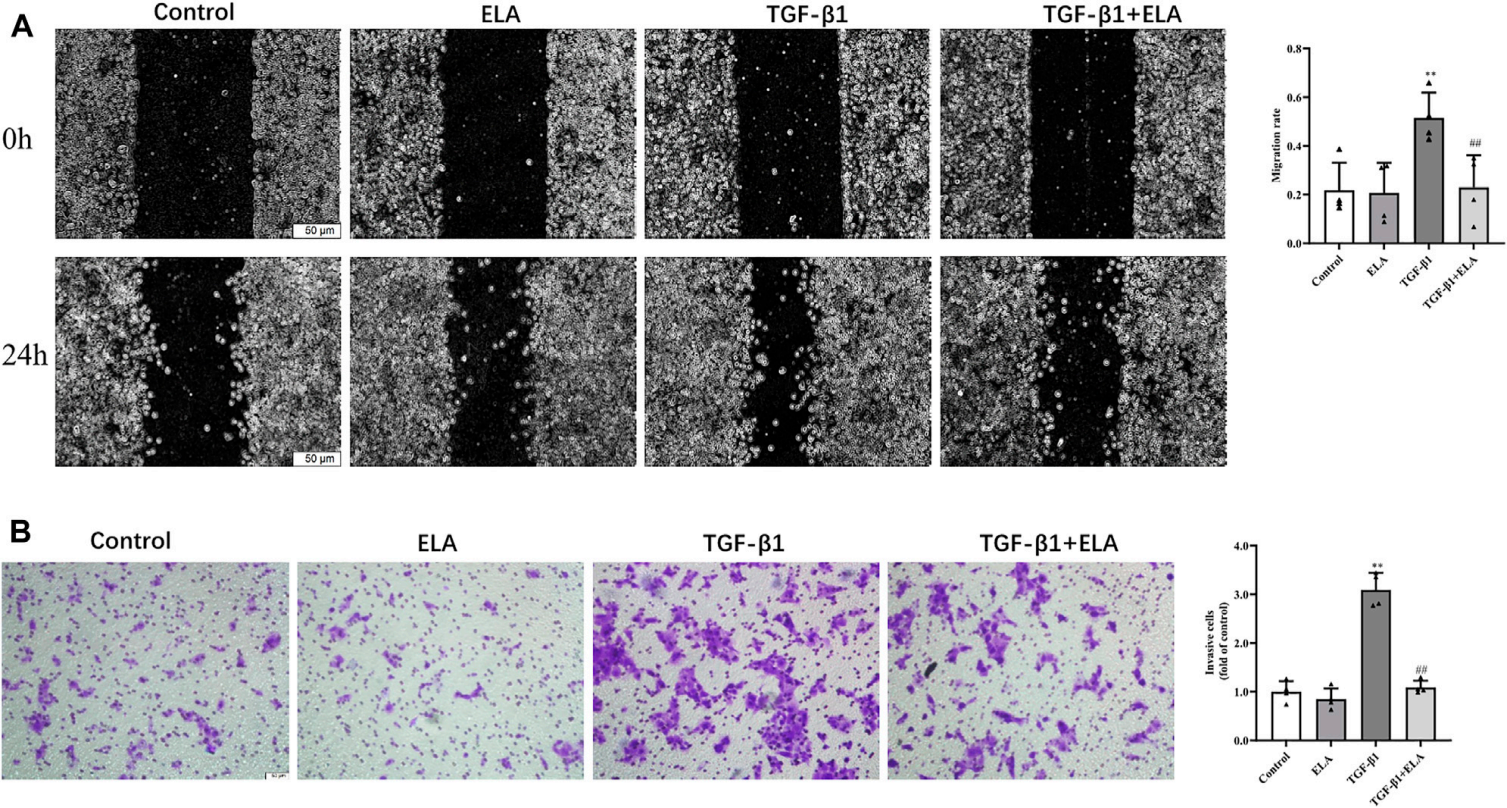
Effects of ELA-32 on TGF-β1-induced migration of HMrSv5. HMrSv5 was divided into four groups according to different treatments: 1) Control group where cells were cultured with DMEM medium containing 10% FBS after 24 h of synchronization; 2) ELA group where cells were treated with ELA-32 (10 μM) for 24 h; 3) TGF-β1 group where cells were treated with TGF-β1 (10 ng/ml) for 24 h; and TGF-β1+ELA group where cells were co-treated with TGF-β1 (10 ng/ml) and ELA-32 (10 μM) for 24 h. Wound healing **(A)** and Transwell **(B)** was observed under optical microscope (scale bar = 50 μm). ImageJ software was used for data statistical analysis. The experiments were repeated four times. Data are expressed as mean ± SD, **p < 0.05 vs. control group; ^##^ *p* < 0.05 vs. TGF-β1 group. Abbreviations: ELA, elabela; TGF-β1, transforming growth factor-β1.

In addition to the recovery of EMT marker expression, ELA-32 treatment also inhibited the TGF-β1-induced increase in migration capacity of the HPMCs. In wound healing assays and Transwell assays, the migration and invasion abilities of HMPCs were increased after administering TGF-β1 (10 ng/ml) but were attenuated on administering ELA-32 (10 μM) (Figure 3). These data suggest that ELA-32 effectively inhibited EMT progress.

### ELA-32 Inhibits the TGF-β1-Induced Activation of the SMAD/ERK1/2/AKT Pathway

**FIGURE 4:**
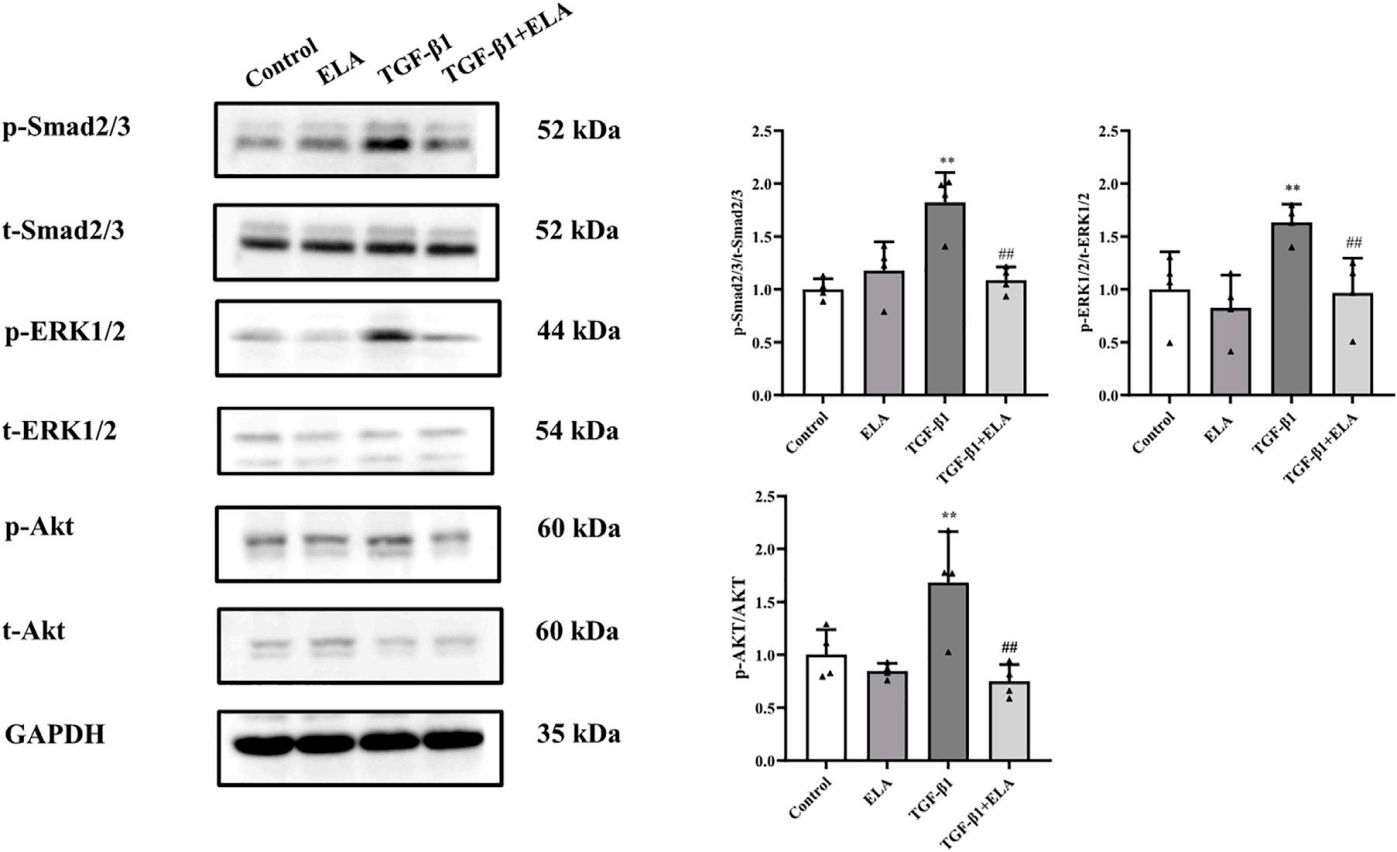
Effects of ELA on TGF-β1-induced activation of the SMAD/ERK/AKT Pathway. HMrSv5 was divided into four groups according to different treatments: 1) Control group where cells were cultured with DMEM medium containing 10% FBS after 24 h of synchronization; 2) ELA group where cells were treated with ELA-32 (10 μM) for 24 h; 3) TGF-β1 group where cells were treated with TGF-β1 (10 ng/ml) for 24 h; and TGF-β1+ELA group where cells were co-treated with TGF-β1 (10 ng/ml) and ELA-32 (10 μM) for 24 h. After stimulate for 24 h, the expression of p-SMAD2/3, p-ERK1/2, and p-AKT were detected by Western blotting. Four independent experiments were carried out in each group. Data are expressed as mean ± SD, **p < 0.05 vs. control group; ^##^ *p* < 0.05 vs. TGF-β1 group. Abbreviations: ELA, elabela; TGF-β1, transforming growth factor-β1.

As the activation of the TGF-β/SMAD pathway is the main cause of PF (Balzer, 2020), we investigated the total SMAD2/3 content and its phosphorylation level in the HPMCs after TGF-β1 treatment to elucidate the mechanism underlying the ELA-32 mediated inhibition of EMT. After TGF-β1 stimulation of HPMCs for 24 h, phosphorylation of SMAD2/3 was increased in the TGF-β1 group, while that of SMAD2/3 was attenuated in the TGF-β1 + ELA group (Figure 4). ELA plays a protective role mainly by activating the ERK1/2 and AKT pathways (Chapman et al., 2021). However, the activation of the AKT and ERK1/2 pathways promotes EMT of HPMCs (Balzer, 2020). Therefore, we examined whether ELA-32 treatment influenced the ERK1/2 and AKT pathways. Interestingly, TGF-β1 treatment enhanced the phosphorylation levels of ERK1/2 and AKT in HPMCs, but these levels decreased after the addition of ELA-32 (Figure 4). These findings indicate that ELA-32 could reduce TGF-β1-induced EMT by suppressing SAMD/ERK/AKT pathway in HPMCs.

## Discussion

**FIGURE 5:**
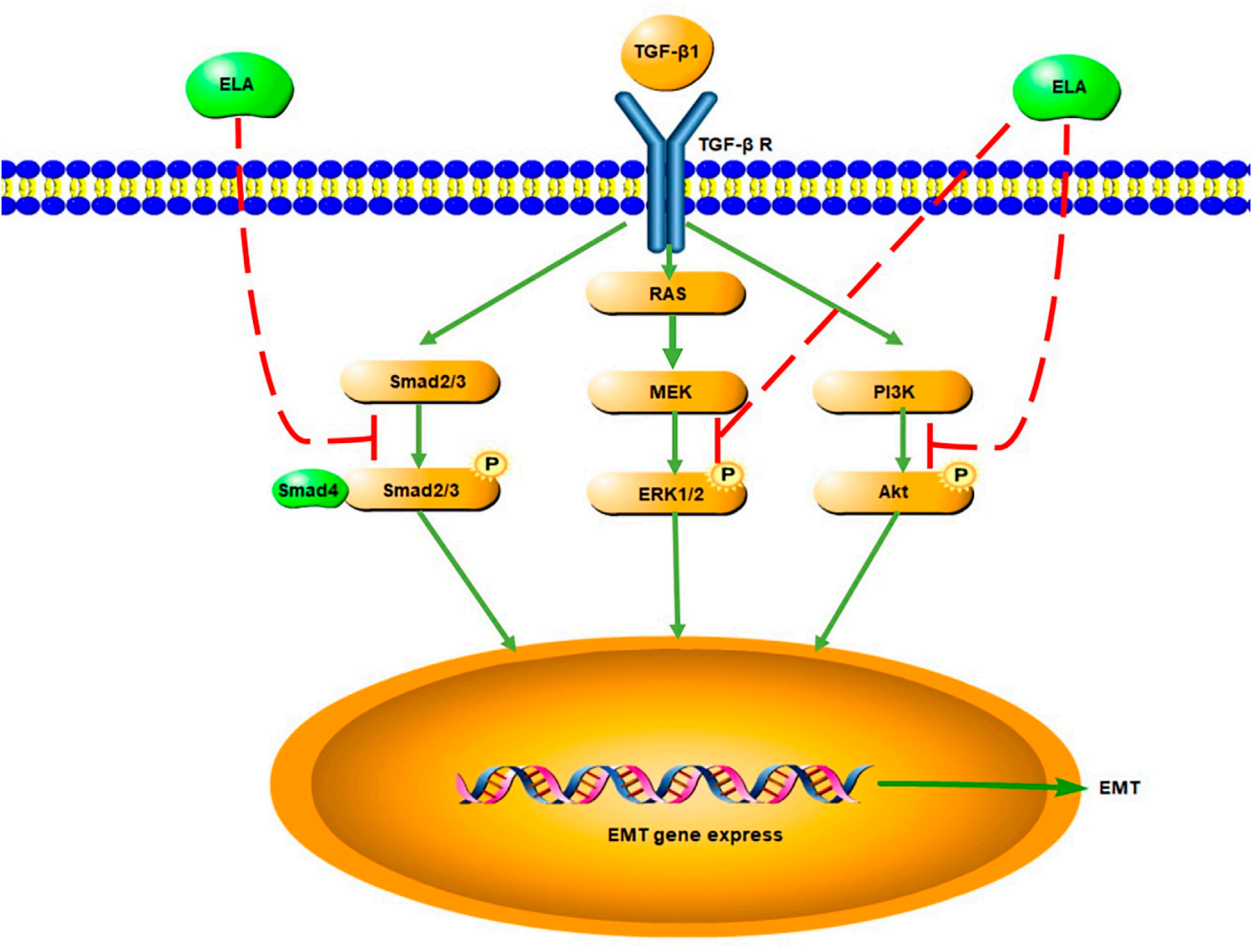
Signaling pathway diagram of ELA inhibiting EMT in HMrSv5.

In this study, we discovered that serum ELA levels increased and serum APLN levels decreased in patients with CKD5 entering the early stage of PD. With the extension of dialysis duration, serum ELA level decreased to the pre-dialysis level, whereas the serum APLN level increased to the pre-dialysis level. Notably, the major risk factor affecting the serum ELA levels in patients undergoing PD was dialysis duration. In addition, serum APLN level positively correlated with dialysis duration. Based on our *in vitro* experimental findings, we found that ELA-32 attenuates the TGF-β1-induced EMT of HPMCs by inhibiting the activation of the TGF-β/SAMD/ERK and AKT pathways (Figure 5).

In the circulatory system, ELA dilates the blood vessels, lowers blood pressure, alters renal blood flow, and increases diuresis (Hus-Citharel et al., 2008; Murza et al., 2016). Since ELA is distributed in the kidney and vascular endothelium in adults, the main factors affecting serum ELA levels are circulatory and renal system diseases. During acute myocardial infarction, compared with healthy people, the serum ELA level of patients showed an acute increase, and it positively correlated with myocardial injury markers troponin I and NT-pro BNP (Dönmez and Acele, 2019). In patients with stable angina pectoris and atrial fibrillation in stable disease, ELA levels will be reduced (Cui et al., 2021; Diakowska et al., 2021). Further, compared with healthy people, serum ELA is lower in patients with diabetic nephropathy (Onalan et al., 2020). In patients with CKD, the serum ELA level gradually decreases with the reduced GFR level, with the lowest values observed in patients with CKD5 (Lu et al., 2020). In our study, we used rGFR to evaluate the residual renal function in patients on PD in comparison with that in patients with CKD5. Our results suggest that after entering the early stage of PD, the serum ELA levels increased in patients with CKD5, corroborating previous results (Lu et al., 2020). Early PD treatment improves residual renal function in some patients with CKD5 (Kuo et al., 2022). We speculate that after patients with CKD5 enter the early stage of PD, the anemia and acidosis of the body will improve to a certain extent, which may promote the transient increase of ELA in the circulatory system. The decrease in peritoneal function leads to the accumulation of uremic toxins in the body, which is accompanied by the continuous aggravation of vascular endothelial damage, which may also lead to the reduction of ELA levels. However, the serum concentration of ELA in patients suggested that with the extension of PD treatment time, patients with PD will lose the protective effect of ELA on the body.

In patients with CKD, the distribution of serum APLN is controversial. Lu et al. (2020) indicated that there is no difference in the serum APLN levels between healthy people and patients with CKD at stages 1–5 without renal replacement therapy. In another study, non-diabetic patients on hemodialysis (HD) had lower serum APLN levels than healthy individuals (Małyszko et al., 2006). On the contrary, the detection of serum APLN level in patients on HD and PD indicated that the level of APLN was higher than that in the normal population (Büyükbakkal et al., 2015; Dogan et al., 2018). The latest detection of serum APLN levels in patients with PD also indicated that APLN levels in this set of patients were higher than those in the normal population (Karaer Büberci et al., 2022). APLN is mainly catabolized by the angiotensin converting enzyme 2. The increase in APLN levels in patients with CKD may be caused by the decrease in the expression of angiotensin converting enzyme 2 due to the uremic microenvironment in patients with CKD that leads to decreased APLN degradation (Wang et al., 2016). The exact role of APLN in EMT is unknown. However, the relationship between APLN and inflammation, which is known to promote EMT (Li et al., 2014), has been studied. Some scholars suggested that APLN can evaluate inflammation and prevent the occurrence of atherosclerotic cardiovascular disease in patients undergoing both PD and HD (Dogan et al., 2018; Trojanowicz et al., 2020; Karaer Büberci et al., 2022). However, some studies have also indicated that APLN induces the expression of inflammatory factors MCP-1, VCAM-1, and ICAM-1 in human umbilical vein endothelial cells by activating the NF-kB/JNK signaling pathway (Trojanowicz et al., 2020). In addition, Sumi et al. (2021) found a weak negative correlation between serum APLN level and dialysis duration, leading them to speculate that APLN promotes peritoneal vascular proliferation. However, in our experiment, the serum APLN levels positively correlated with dialysis duration in patients on PD, contrary to the findings of Sumi et al. (2021). Furthermore, the other factors involved still remain unclear. Therefore, more studies are needed to clarify the role of APLN in EMT.

As the key cytokine-promoting PD-associated PF, TGF-β plays an important role in promoting fibrosis through the classical SMAD2/3 and non-classical ERK1/2 and AKT pathways (Balzer, 2020). First, we chose to use TGF-β1 to establish an EMT model of HPMCs, and then administered ELA-32 (10 μM) treatment. Our results found that ELA-32 could treat TGF-β1-induced EMT of HPMCs from the aspects of morphology and protein expression. The ELA-mediated activation of the PI3K/AKT pathway produces protective effects in kidney disease (Dagamajalu et al., 2022). However, the activation of this pathway promotes the occurrence of PF in the peritoneal tissues of patients on PD. Notably, our *in vitro* results showed that it did not have a significant effect on activating ERK1/2 and AKT in HPMCs in the ELA-32 treated group. On the contrary, ELA-32 could reduce TGF-β1-induced EMT by suppressing the SAMD/ERK/AKT pathway in HPMCs. Hence, we suggested that ELA-32 may be added as a protective peptide to peritoneal dialysate during PD; when absorbed into the blood through the peritoneum, it may improve the state of ELA deficiency in late PD. ELA-32 may be a potential therapeutic target for future studies on the prevention and treatment of PD-related PF.

However, the current study had some limitations. First, due to the small sample size, the results were limited, which mandates further studies with a large sample size. Second, other factors that influence the serum levels of ELA and APLN in patients on PD need to be further explored. Third, we mainly detected the serum levels of ELA and APLN in patients without assessing whether ELA and APLN were expressed in the dialysate effluent and peritoneal tissue of patients on PD during different dialysis time periods and whether the two were consistent with the serological test results. Finally, the effect of ELA-32 in inhibiting the EMT of HMPCs was observed *in vitro*; further *in vivo* experiments are necessary to verify the results.

In conclusion, our findings demonstrated that the serum ELA level decreased in patients receiving PD after prolonged dialysis time. Furthermore, ELA-32 effectively inhibited the TGF-β1-induced EMT of HPMCs by inhibiting the TGF-β/SMAD/ERK and AKT signaling pathways.

## Data Availability

The original contributions presented in the study are included in the article/Supplementary Material, further inquiries can be directed to the corresponding author.
